# Exclusion of Medicare Advantage Enrollees From Medicare Health Outcomes Analyses: Potential for Bias

**DOI:** 10.1093/geroni/igaa057.557

**Published:** 2020-12-16

**Authors:** Arseniy Yashkin, Igor Akushevich, Anatoliy Yashin

**Affiliations:** Duke University, Durham, North Carolina, United States

## Abstract

The aim of this paper is to assess the extent of the potential bias introduced by the exclusion of the Medicare Advantage (MA) population– an increasingly sizeable (31% of all beneficiaries in 2017) subset of the Medicare population which does not provide claims data to the Centers for Medicare and Medicaid Services– from Medicare-based health outcomes and epidemiologic analyses. Using self-reported data from the Health and Retirement Study together with monthly information on Medicare enrollment, we compared MA enrollees with individuals enrolled in traditional Medicare (TM) on 42 variables representing demographic, socioeconomic, adverse health behavior and health status-related characteristics over the 1991-2015 period. We used both univariate analysis (t-tests and standardized differences) and multivariate logistic regression to compare the two groups. We found that apart from differences in economic (higher in TM group) and education status (lower in MA group) – which have been increasing in magnitude over the 1991-2015 period– the MA subset was highly comparable with the traditional Medicare (TM) population. Even though the TM population was characterized by slightly higher levels of morbidity, the resulting crude prevalence rates for common age-related diseases in the TM/MA groups were within each other’s 95% confidence intervals and did not represent a major source of bias. MA membership was not associated with increased mortality at any point over the 1991-2015 period. We conclude that exclusion of the MA population will not lead to notable bias in health outcome analyses apart from those for which income and education are important explanatory factors.

